# Guidance for Evidence-Informed Policies about Health Systems: Linking Guidance Development to Policy Development

**DOI:** 10.1371/journal.pmed.1001186

**Published:** 2012-03-13

**Authors:** John N. Lavis, John-Arne Røttingen, Xavier Bosch-Capblanch, Rifat Atun, Fadi El-Jardali, Lucy Gilson, Simon Lewin, Sandy Oliver, Pierre Ongolo-Zogo, Andy Haines

**Affiliations:** 1 McMaster Health Forum, Centre for Health Economics and Policy Analysis, Department of Clinical Epidemiology and Biostatistics, and Department of Political Science, McMaster University, Hamilton, Ontario, Canada; 2 Norwegian Knowledge Centre for the Health Services, Oslo, Norway; 3 Swiss Tropical and Public Health Institute, Basel, Switzerland; 4 Global Fund to Fight AIDS, Tuberculosis and Malaria, Geneva, Switzerland and Imperial College, London, United Kingdom; 5 Department of Health Policy and Management, American University of Beirut, Beirut, Lebanon and McMaster Health Forum, McMaster University, Hamilton, Ontario, Canada; 6 University of Cape Town, Cape Town, South Africa, and London School of Hygiene and Tropical Medicine, London, United Kingdom; 7 Norwegian Knowledge Centre for the Health Services, Oslo, Norway, and Health Systems Research Unit, Medical Research Council of South Africa, Cape Town, South Africa; 8 EPPI-Centre, Social Science Research Unit, Institute of Education, London, United Kingdom; 9 Centre for the Development of Best Practices in Health, Yaoundé Central Hospital, and University of Yaoundé, Yaoundé, Cameroon; 10 London School of Hygiene and Tropical Medicine, London, United Kingdom

## Abstract

In the second paper in a three-part series on health systems guidance, John Lavis and colleagues explore the challenge of linking guidance development and policy development at global and national levels.

**Summary Points**

- Contextual factors are extremely important in shaping decisions about health systems, and policy makers need to work through all the pros and cons of different options before adopting specific health systems guidance.
- A division of labour between global guidance developers, global policy developers, national guidance developers, and national policy developers is needed to support evidence-informed policy-making about health systems.
- A panel charged with developing health systems guidance at the global level could best add value by ensuring that its output can be used for policy development at the global and national level, and for guidance development at the national level.
- Rigorous health systems analyses and political systems analyses are needed at the global and national level to support guideline and policy development.
- Further research is needed into the division of labour in guideline development and policy development and on frameworks for supporting system and political analyses.
- This is the second paper in a three-part series in *PLoS Medicine* on health systems guidance.

*This is one paper in a three-part series that sets out how evidence should be translated into guidance to inform policies on health systems and improve the delivery of clinical and public health interventions.*

## Introduction

Policies about health systems can have profound impacts on citizens, patients, health professionals, and managers. For physicians, for example, the impacts can include changing their scope of practice (a governance arrangement), how they are paid (a financial arrangement), where they provide care (a delivery arrangement), and how their practices are supported in providing the types of care that citizens and patients value (an implementation strategy). Contextual factors are extremely important in shaping decisions about health systems, and policy makers have to work through all the pros and cons of different options before developing new policies on health systems.

In this paper, which is the second of a three-part series on health systems guidance [1],[2], by considering issues raised during meetings of the World Health Organization's (WHO) Task Force on Developing Health Systems Guidance (Box 1), we:

- Explore the links between health systems guidance development and policy development at global and national levels;
- Examine the range of factors that can influence policy development.

The first article in the series makes a case for developing guidance to inform decisions on health systems-level questions based on an analysis of strategic health sector documents, explores specific challenges in producing such guidance, and identifies options for addressing these challenges [1]. The third paper focuses on assessing how much confidence can be placed on health systems research evidence in both guidance and policy development processes [2].

**Box 1. The Task Force on Developing Health Systems Guidance**

To improve how it responds to requests for guidance on health systems, WHO established the Task Force on Developing Health Systems Guidance in 2009. Briefly:

- WHO selected the 20 members of this Task Force to ensure diversity in terms of skills and experience in four broad domains—health policy and systems research (30% of the panel members), systematic reviews (55%), national deliberative processes (20%), and guidance development (40%)—and in terms of gender (25% female) and current base in a low- and middle-income country (30%).
- The Task Force provided input to the development of a *Handbook for Developing Health Systems Guidance* and to the identification of broader issues that warranted further dialogue and debate [12].
- As part of this process, the Task Force reviewed approaches to developing clinical guidelines and the instruments used as well as the broader literature on the four broad domains listed above, which were synthesized by the Handbook developers.
- The Task Force suggested ways in which some of the approaches and instruments used to develop clinical guidelines could be adapted for use in the development of health systems guidance and indicated where there were important differences between these two types of guidance.

The writing group for this paper further considered the issues raised in these discussions and produced a first draft of the manuscript for comment by the Task Force. This paper and the other two in the series [1],[2] were finalised after several iterations of comments by the Task Force and external reviewers.

Here and in the other two papers in the series, we rely on a set of key definitions (Text S1). While the definitions of health systems and health systems interventions may be familiar to many, the definition of health systems guidance differs significantly from the definition of clinical guidelines [3], both in its focus on including options for consideration and in its focus on using guidance to assist decision-making in a range of settings. The importance of contextual factors in shaping decisions about health systems means that health systems guidance should include information about what is known about the pros and cons of different options, the factors that will likely influence decisions about the options in different settings, and the tools that can support local guidance or policy development processes. A policy brief can be used to provide background evidence to inform a policy dialogue among stakeholders [4],[5], which can in turn result in the articulation of the preferred policy option(s).

Importantly, in our definitions of both health systems guidance and policy briefs (Text S1) and throughout this paper, we emphasize the importance of being systematic and of involving all stakeholders. The recent World Health Assembly resolution on guidance for health system policies gives much needed attention to the issue of health systems guidance [6],[7]. However, the resolution contains only two uses of the phrase “evidence-based” (one for the assessment of a country's health and health system problems, the other for responses to evolving problems) and one call for “involv[ing] all relevant stakeholders.” Moreover, the resolution and related materials are largely silent on the need to follow systematic processes for evidence synthesis and stakeholder engagement.

## Links between Guidance Development and Policy Development

**Figure 1:**
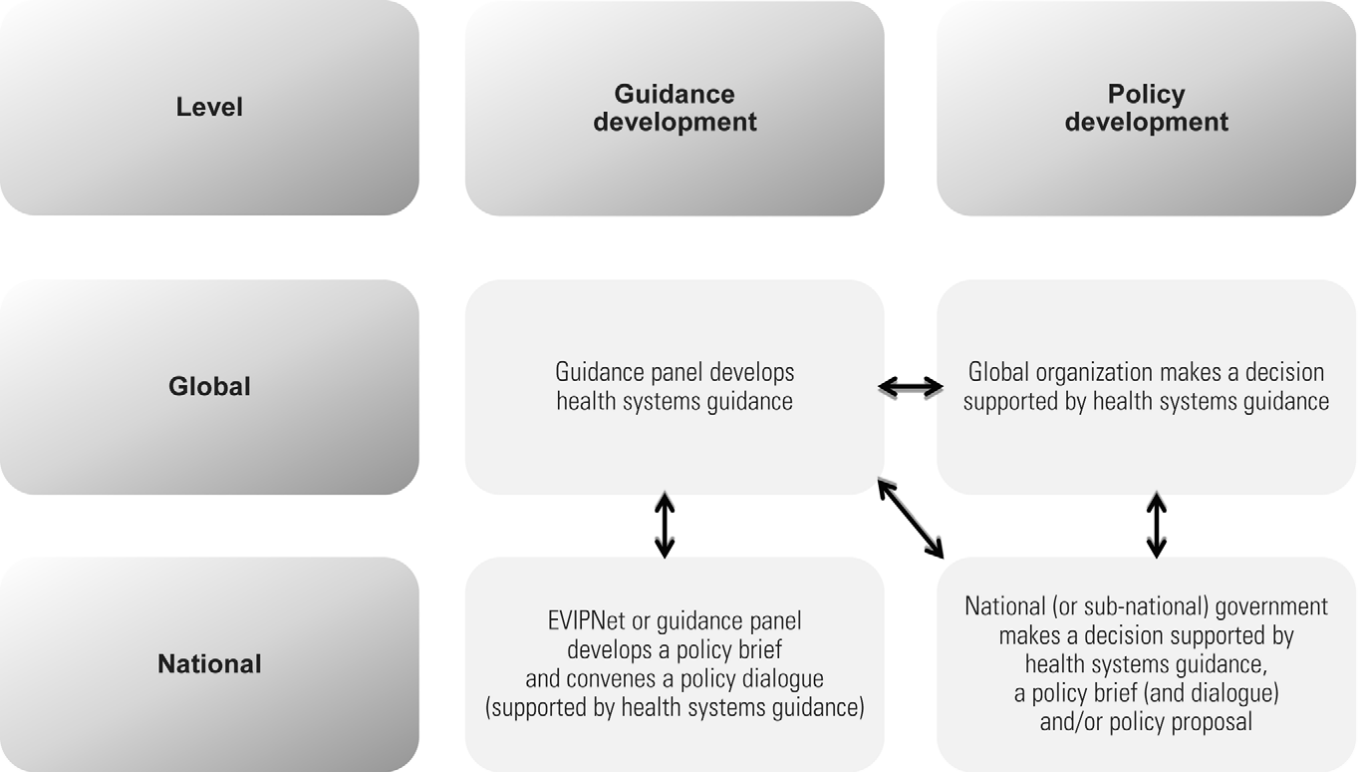
Potential links between guidance and policy development at global and national levels.

Economy of scale and efficiency considerations at the global level, and resource and capacity constraints at the national level, mean that a division of labour among global guidance developers, global policy developers, national guidance developers, and national policy developers is needed to support evidence-informed policy-making about health systems (Figure 1). All these groups would help to set priorities and provide feedback (double-headed arrows in Figure 1).

The first group—a panel charged with developing guidance about health systems at the global level similar to the panels convened by WHO to address specific issues—could likely best add value by developing health systems guidance and by supporting its use in three types of processes:

- Policy development at the global level (where applicable);
- Guidance development at the national level;
- Policy development at the national level.

### Policy Development at the Global Level

International organizations like WHO and the World Bank, global initiatives like the Global Alliance for Vaccines and Immunization and the Global Fund to Fight AIDS, Tuberculosis and Malaria, and many bilateral initiatives and some very large multinational nongovernmental organizations (NGOs) could draw on the global guidance to inform decisions within their respective remits. Such decisions may be about how the organization invests its money directly (e.g., bulk-purchasing arrangements) or about how it attaches conditionalities to funds invested in related areas (e.g., staffing in HIV clinics). For some of these organizations and initiatives, making policy decisions about health systems is a relatively new area that lies outside their normally perceived remit, but the impacts of their involvement in making policy decisions on health systems could be profound given their scale of funding.

### Guidance Development at the National Level

A national Evidence-Informed Policy Network (EVIPNet, see Text S1) sponsored by government or a guidance panel appointed by government (or other groups provided their members have the skills and experiences for such work) could draw on the global guidance to develop a country-specific policy brief that contextualizes the health system problem, options for addressing the problem, key implementation considerations, and (possibly) monitoring and evaluation considerations. The policy brief could draw on context-specific data and research evidence in each of these domains and could take into consideration any global decisions that might be considered binding on the country. A recent example of such an effort is the policy briefs that were developed to scale up the widespread use of artemisinin-based combination therapy to treat malaria. In this instance, global guidance spurred (but did not fully support) the national guidance development processes that EVIPNets undertook in several African countries and regions [8].

An EVIPNet could also convene a policy dialogue that allows the data and research evidence contained in the policy brief to inform and be considered alongside the views, experiences, and tacit knowledge of those who will be involved in, or affected by, future decisions about the health system problem [5]. The evidence brief and dialogue summary could then be used by the national government to inform a decision.

Global decisions that might be considered in national guidance development processes include the normative standards that WHO member states collectively endorse at the World Health Assembly and agree to abide by in their respective countries. However, worryingly, a review of the statements made by the World Bank and WHO on topics addressed by the World Health Assembly between 2000 and 2003 (including three broad health systems topic areas) revealed that these statements are rarely consistent with the direction and the nature of effect claims from systematic reviews, which suggests that there is significant potential for improvement in how these organizations use or report the use of synthesized research evidence in their policy-development processes [9].

### Policy Development at the National Level

A national government could make decisions for its country by drawing on the health systems guidance produced at the global level and/or a policy brief produced at the national level (with or without the summary of a policy dialogue that was informed by the policy brief) and by keeping in mind any global decisions that it acknowledges as binding on itself. The national government may choose to convene one or more policy dialogues or use other stakeholder-engagement approaches to support its decision-making and to reach agreement among stakeholders, ideally informed by the policy brief and supported by relevant tools highlighted in the guidance produced at the global level.

### Will This Proposed Division of Labour Work?

We suggest that this proposed division of labour should be explored retrospectively by looking at several cases where health systems guidance has been developed at the global level and examining how the process could have unfolded differently with a view to refining the process. The division of labour could also be examined prospectively by examining cases where guidance is being developed at the global level and EVIPNets (among others) are drawing on this guidance to develop national policy briefs and convene national policy dialogues. Additional insights could be derived by examining the lessons learned in the field of health technology assessment where a similar need for a division of labour has been documented, albeit mainly at the level of drugs, devices, and other technologies [10].

## Factors That Can Influence Policy Development

The final stage in the process of health systems policy development is the preparation of a document (or documents) produced within a national government to support decision-making by that government. A range of names might be applied to the document(s), which is ideally informed by global guidance, a national policy brief, and other inputs, but for simplicity, we refer to this decision-support document as a policy proposal.

The health systems guidance (which emphasizes global evidence), policy brief (which emphasizes global and national research evidence), and policy proposal (which emphasizes the many considerations supporting a preferred course of action) all need to synthesize what is known and not known about the pros and cons of the options under consideration. They also all need to assess the factors that can influence the choice, implementation, and monitoring and evaluation of these options in different settings (or in the case of the policy brief and policy proposal, at least refer to the synthesis contained in the guidance and policy brief, respectively, when they exist). While varying in emphasis, each of these documents would ideally document:

- Key features of an assessment about how to address a health system problem;
- Key features of a health system (or health systems) that can influence decision-making about how to address a health system problem;
- Key features of a political system (or political systems) that can influence decision-making about how to address a health system problem.

### Key Features of an Assessment about How to Address a Health System Problem

An assessment about how to address a health system problem requires working through the underlying problem, the appropriate options to address the problem, and implementation issues (see Table S1). Much of what is known and not known about each of these areas can be derived from available data and research evidence. Health systems guidance that is produced at the global level can present overall summaries of the answers to these questions and identify patterns in the variation in these answers across health systems and political systems. Synthesized research evidence about health systems is increasingly available, and initiatives to make it easier to find and use evidence can support guidance development at the global level (e.g., Health Systems Evidence at http://www.healthsystemsevidence.org, which is now available in Arabic, Chinese, English, French, Portuguese, Russian, and Spanish). A policy brief produced at the national level can supplement the data and research evidence contained in global health systems guidance with local data and research evidence in order to present as clearly as possible what is known about how to address a health system problem in a particular country.

### Key Features of Health Systems That Can Influence Decision-Making about How to Address a Health System Problem

An assessment of the key features of health systems that can influence decision-making about how to address a health system problem involves working through existing governance, financial, and delivery arrangements to determine which arrangements might help or hinder any options being considered (Table 1). A new health systems intervention such as pay-for-performance might “fit” into one health system, but the same intervention or part of the health system might require significant adjustment or complementary interventions to fit into a system where performance data are not collected systematically. Moreover, any new health systems intervention may have unanticipated consequences for the existing health system arrangements in which it is introduced, for example, by removing incentives for some types of activity.

**Table 1:** Key features of a health system that can influence decision-making about how to address a health system problem.

Key Features	Examples
**Governance arrangements**	
• Policy authority	• National ministry sets policy directions for the health system but sub-national (e.g., provincial) ministries and private organizations can accept, adapt, or reject them• National and provincial ministries only weakly enforce anti-corruption policies
• Organizational authority	• Private for-profit companies own most hospitals in urban centres, whereas religious charities own most hospitals in rural areas• A national network of pharmacies acts as a de facto monopoly
• Commercial authority	• Limited regulation of patents, prices, and marketing of diagnostic tests• Strong safeguards against the production and sale of counterfeit medicines
• Professional authority	• Only physicians have the regulatory authority to diagnose and prescribe• Mandatory continuing professional development of health professionals
• Consumer and stakeholder involvement	• Half of the seats on all health system advisory councils are reserved for consumers• Large non-governmental organizations participate in key ministry planning meetings
**Financial arrangements**	
• Financing systems	• Mandatory participation in a private or community-based insurance scheme• Reliance on donor contributions for major infectious disease programs but insufficient funding from any source for non-communicable disease programs
• Funding organizations	• Ministry uses global budgets to fund public and private not-for-profit hospitals• Clinics incur a financial penalty if they fail to achieve performance targets, one of which is high consumer satisfaction ratings
• Remunerating providers	• All hospital-based personnel are paid by salary• Community health workers receive a bonus if they achieve performance targets
• Purchasing products and services	• List of substitutable products and services is updated every three years• Prior approval requirements are in place for high-cost purchases
• Incentivizing consumers	• Patients face large out-of-pocket costs for seeking care outside their local clinic• Patients receive conditional cash transfers for select health-related behaviours
**Delivery arrangements**	
• How care is designed to meet consumers' needs	• Local cultural beliefs limit the demand for certain types of programs and services• Optimal packages of care (e.g., Integrated Management of Childhood Illness) have been adapted to the country and are widely used
• By whom care is provided	• Many parts of the country are experiencing physician shortages• Community health workers receive high-quality training and supervision to play a defined role in tuberculosis control
• Where care is provided	• Hospitals in urban areas have high-quality infrastructure• Clinics frequently lack functioning diagnostic equipment and covered/reimbursed medicines
• With what supports is care provided	• Information and communication technologies do not function well in rural and remote communities• Quality monitoring and improvement systems are in place and functioning well

The taxonomy is drawn from Health Systems Evidence (http://www.healthsystemsevidence.org), which is an adapted and more detailed operationalization of the WHO “building blocks of health systems” [15], and the examples are drawn from a range of sources (e.g., [21]). The word “care” within the section of delivery arrangements could be replaced by programs and services or by prevention, treatment, and support when the focus is more on public health than on clinical care.

Some of what is known and not known about the key health system arrangements can be derived from available data and research evidence. Health systems guidance that is produced at the global level can present overall summaries of what is known about which arrangements are key for achieving the desired impacts in different health system contexts. A policy brief produced at the national level can enrich these summaries with local data and research evidence in order to present as clearly as possible what is known about how existing health system arrangements may influence the selection and implementation of options. In this way, policy-making about health systems can be informed by a good understanding of the system-level context for an option and the range of its potential desired and undesired system-wide effects, and any adaptation and re-design of the option (and potentially other health system arrangements) that is needed to optimize synergies among health system elements.

### Key Features of Political Systems That Can Influence Decision-Making about How to Address a Health System Problem

An assessment of the key features of a political system that can influence decision-making about how to address a health system problem involves working through the institutions, interests, and ideas that currently drive decision-making, as well as the “external factors” that can open windows of opportunity to introduce change (Table 2). As above, globally produced health systems guidance can ideally present overall summaries about what is known about which features are key and identify patterns in the variation of these features across health systems and political systems. A policy brief produced at the national level can enrich the data and research evidence from health systems guidance with local data and research evidence. Moreover, a policy dialogue conducted at the national level can further enrich the available data and research evidence with local views, experiences, and tacit knowledge about how the political system really works and which system features are most important for the issue at hand.

**Table 2:** Key features of a political system that can influence decision-making about how to address a health system problem.

Key Features	National (or Sub-National) Examples
**Institutions**	
• Government structures	• Constitution states that health care is a sub-national responsibility, so provincial finance and health ministries are where most key decisions are made• Health minister has delegated authority from the prime minister and cabinet to make almost all key decisions regarding the health system
• Policy legacies	• Legislation created only a limited role for the ministry of health so civil servants never developed the administrative capacities required to pursue many options• Health care insurance policy has shaped the thinking and influence of the country's medical association
• Policy networks	• A standing government-appointed guidance panel engages key stakeholders in the process of informing policy-making on select issues• A committee comprised of government and medical association representatives makes many recommendations that later become law
**Interests**	
• Interest groups	• For-profit companies that face concentrated benefits or costs in relation to an option mobilize quickly and exert pressure effectively• Nursing association has the technical and communication staff needed to influence the policy-making process
• Civil society	• Citizens are poorly organized and groups representing them have difficulty reaching consensus on their preferred option• Lack of independent media hampers dialogue and debate
**Ideas**	
• Values	• Widely held values support a focus on equity in the health systems• Government holds a strong pro-market orientation
• Personal experiences	• Personal experiences of the minister influence much of her decision-making• A highly visible consumer representative very effectively mobilizes the stories of individuals' poor treatment in the system to push for change
• Research evidence	• A systematic review suggests that one option is more effective and cost-effective than others• A qualitative synthesis identified that stakeholders' views and experiences are such that one option is likely to achieve higher coverage rates than others
**External factors**	
• Political change	• Election brings a new president or legislative coalition to power• Cabinet shuffle introduces a new minister to the health portfolio
• Economic change	• Global economic crisis reduces donors' capacity to support national programs• National economic situation spurs calls to “do more with less”
• Release of major reports	• A report by a prominent international organization endorses one option over others• An external audit of a malaria eradication program reveals significant corruption
• Technological change	• Mobile phone technology introduces new possibilities for performance management
• New diseases	• An influenza outbreak spreads rapidly to other countries
• Media coverage	• A series of investigative news articles in the national newspaper reveals the weak enforcement of contracts in the health system

The framework is adapted from one presented elsewhere [22], which in turn was informed by a set of related frameworks (e.g., [23],[24]) as well sub-frameworks (e.g., [25]–[28]).

### How Do These Assessments Relate to One Another and to Other Approaches?

These three types of assessment are clearly interrelated. For example, empirical research has shown that an option is often deemed an appropriate solution if it is technically feasible (which can come from Tables S1 and 1), fits with dominant values and the current national/provincial mood (which can come from Table 2), and is acceptable in terms of affordability (which can come from Tables S1 and 2) and likely political support or opposition (which can come from Table 2) [11]. The *Handbook for Developing Health Systems Guidance* sets out an approach that focuses on the intervention as tested (e.g., costs, appropriateness, ease of implementation), requirements in relation to implementers or facilitators (e.g., credibility, skills, experience), requirements in terms of users of the interventions (e.g., capacity, training), and factors related to the context (e.g., political, socioeconomic, rights), which again can be traced to Tables S1, 1, and 2 [12]. Unpacking these assessments further, as we have done here, can help to provide a more systematic and transparent assessment.

As we discuss in the next paper, existing approaches to grading the quality of recommendations about clinical options will likely require significant modification for use in a health system context [2]. The GRADE approach [13], for example, focuses only on two of the system-level factors that we have described—“values and preferences” and “feasibility”—but there are many more factors that will influence the choice of options for addressing a health system problem in different settings (as well as the implementation and the monitoring and evaluation of the preferred option). Also, the GRADE approach is typically executed by experts, and, while it can inform and complement policy dialogues and other stakeholder-engagement processes, it cannot substitute for these processes in the assessment of system factors.

## Conclusions

Our proposed division of labour links guidance development at the global level with policy development at the national level. Balancing a broad range of system and political considerations to come to a reasoned judgment about how to address a health system problem is arguably the purview of those who have been given the accountability to make decisions about health systems, whether democratically elected or appointed. Typically, these policy makers are located at the national level. Our examination of the range of factors that can influence policy development (and that can be flagged for consideration in health systems guidance) highlights the need for rigorous system and political analyses in policy briefs at the national level. These analyses can be supported at the global level by health systems guidance that presents overall summaries about what is known about which health and political system features help and hinder particular options. These analyses can also be aided by the frameworks presented in Tables S1, 1, and 2, by the SUPporting POlicy Relevant Trials (SUPPORT) tools [14]—a set of tools that can be used by people involved in finding and using research evidence to support evidence-informed health policy-making—and by a range of other approaches such as the evidence synthesis and grading approaches presented in the other two papers in the series [1],[2] and the decision trees, system modeling, and evaluation frameworks presented in the *Handbook for Developing Health Systems Guidance* [12]. Those involved in policy development at the national level will need to, as representatives of member states of WHO, push for change at the global level if guidance development in the area of health systems is to support the type of context-sensitive policy development at the national level that we have proposed here.

Certainly, there is a fruitful research agenda ahead for those interested in studying the division of responsibilities across guidance panels at the global level, EVIPNets or guidance panels at the national level, and national (or sub-national) governments, as well as the conditions under which global organizations have a legitimate role in making decisions about health systems. A complementary research agenda could focus on assessing frameworks and approaches to supporting system and political analyses, particularly in the difficult (but not uncommon) situation where a health system intervention is actually a complex bundle of interventions that can interact in helpful and unhelpful ways. The results of such research could inform ongoing modifications to the division of labour that we propose in this paper to ensure that limited resources are used wisely and that both the best available research evidence and the contextual insights of key stakeholders informs guidance development and policy development.

## Supporting Information

Alternative Language Summary Points S1 **Translation of the Summary Points into Spanish by Xavier Bosch-Capblanch**
(DOC)

Alternative Language Summary Points S2 **Translation of the Summary Points into French by Bruno Clary, William Lenoir, and Lise Beck**
(DOC)

Alternative Language Summary Points S3 **Translation of the Summary Points into Portuguese by Bruno Viana**
(DOC)

Alternative Language Summary Points S4 **Translation of the Summary Points into Arabic by Fadi El-Jardali**
(DOC)

Table S1 **Key features of an assessment about how to address a health system problem**
(DOC)

Text S1 **Definitions used in this paper**
(DOC)

## Abbreviations

EVIPNet: Evidence-Informed Policy Network
WHO: World Health Organization
