# Supplementary material for: Guidance for Evidence-Informed Policies about Health Systems: Linking Guidance Development to Policy Development
Source: PLoS Med. 2012 Mar 13;9(3):e1001186. doi: 10.1371/journal.pmed.1001186 (PMC3302830; doi:10.1371/journal.pmed.1001186)
Supplement: Alternative Language Summary Points S4 — Translation of the Summary Points into Arabic by Fadi El-Jardali (DOC) [file pmed.1001186.s004.doc]

Guidance for Evidence-Informed Policies about Health Systems: Linking Guidance Development to Policy Development

**نقاط موجزة**

- إن عوامل البيئة المحيطة تكتسب أهمية قصوى في تشكيل القرارات المتعلقة بالأنظمة الصحية، وعلى صانعي القرار الأخذ بكافة السلبيات والإيجابيات للاحتمالات المختلفة، قبل تبني مشورة معينة بخصوص الأنظمة الصحية.

- هناك حاجة إلى تقسيم المهام بين الفرق الدولية/العالمية المتخصصة بتطوير المشورة وتلك المتخصصة بتطوير السياسات، وبين نظرائها من الفرق المحلية، بهدف دعم عملية صناعة القرار المستندة إلى البيّنات في القرارات والسياسات المتعلقة بالأنظمة الصحية.

- يمكن للجنة دولية من الخبراء المعنيين بتطوير إرشادات الأنظمة الصحية على المستوى العالمي أن يعطوا قيمة مضافة لعملهم من خلال التأكد من صلاحيته للاستخدام في تطوير السياسات على المستويات المحلية إلى جانب المستوى الدولي/ العالمي، وأنه أيضاً يصلح لتقديم المشورة على المستويات المحلية.

- هناك حاجة لتحاليل جادة ومعمّقة للأنظمة الصحية والسياسية على المستويات المحلية والدولية، لدعم آليات وضع وتقديم المشورة والسياسات.

- هناك حاجة إلى المزيد من الأبحاث حول توزيع الأدوار في وضع وتطوير المشورة والسياسات، وحول أطر العمل الداعمة في مجال تحليل الأنظمة والتحليل السياسي.
