# Supplementary material for: Guidance for Evidence-Informed Policies about Health Systems: Linking Guidance Development to Policy Development
Source: PLoS Med. 2012 Mar 13;9(3):e1001186. doi: 10.1371/journal.pmed.1001186 (PMC3302830; doi:10.1371/journal.pmed.1001186)
Supplement: Table S1 — Key features of an assessment about how to address a health system problem (DOC) [file pmed.1001186.s005.doc]

**SI2: Key features of an assessment about how to address a health system problem**

| **Key features** | **Questions that can be answered by data and research evidence** |
| --- | --- |
| Health system problem | 1. What is the **problem**? |
|  | 1. How did the problem come to **attention** and has this process influenced the prospect of it being addressed? |
|  | 1. What **indicators** can be used, or collected, to establish the magnitude of the problem and to measure progress in addressing it? |
|  | 1. What **comparisons** can be made to establish the magnitude of the problem and to measure progress in addressing it? |
|  | 1. How can a problem be **framed** (or described) in a way that will motivate different groups? |
| Options for consideration | 1. Has an appropriate set of **options** been identified to address the problem? |
|  | 1. What **benefits** are important to those who will be affected and which benefits are likely to be achieved with each option? |
|  | 1. What **harms** are important to those who will be affected and which harms are likely to arise with each option? |
|  | 1. What are the local **costs** of each option and is there local evidence about their **cost-effectiveness**? |
|  | 1. What **adaptations** might be made to any given option and might they alter its benefits, harms and costs? |
|  | 1. Which stakeholders’ **views and experiences** might influence the acceptability of an option and its benefits, harms, and costs? |
| Implementation considerations | 1. What are the potential **barriers** to the successful implementation of the policy? |
|  | 1. What strategies should be considered in order to facilitate the necessary behavioural changes among **patients/citizens**? |
|  | 1. What strategies should be considered in order to facilitate the necessary behavioural changes among **health workers**? |
|  | 1. What strategies should be considered in order to facilitate the necessary **organizational** **changes**? |
|  | 1. What strategies should be considered order to facilitate the necessary **system** **changes**? |

The questions are drawn from the SUPPORT tools series [18-20]
