# Supplementary material for: Guidance for Evidence-Informed Policies about Health Systems: Linking Guidance Development to Policy Development
Source: PLoS Med. 2012 Mar 13;9(3):e1001186. doi: 10.1371/journal.pmed.1001186 (PMC3302830; doi:10.1371/journal.pmed.1001186)
Supplement: Text S1 — Definitions used in this paper (DOC) [file pmed.1001186.s006.doc]

**SI1: Definitions used in this paper**

| - Health systems: consists of all organizations, people and actions whose primary intent is to promote, restore or maintain health”[15] - Health systems interventions: establishment or modifications to governance, financial and delivery arrangements within health systems, as well as implementation strategies within health systems, the focus of which is to strengthen health systems in their own right or to get cost-effective programs, services and technologies (e.g., drugs, vaccines and diagnostic tests) to those who need them.   - Such interventions can be distinguished from clinical interventions (e.g., antenatal visits and drugs) and from public health interventions (immunizations and health promotion campaigns) - Health systems guidance: systematically developed statements created at the global or national level to assist decisions about options for addressing a health system problem in a range of settings and to assist with implementation and with monitoring and evaluation   - Health systems guidance could take many forms, but one example could be statements such as: ‘it would be reasonable to pursue option A to address health system problem B in circumstances such as those faced in countries C and D, taking into account health system features E and F and political system features G and H, whereas it would be less reasonable to pursue option X in such countries given....’   - In the absence of research evidence, health systems guidance could identify the nature of any monitoring and evaluation that would be needed.   - A health systems analysis (Table 1) and a political analysis (SI2) would be needed in addition to the assessment of the health system problem under consideration - Policy brief (evidence brief[4]): systematically developed statements created at the national or sub-national level to assist decisions about appropriate options for addressing a health system problem in that specific setting that may also assist with implementation and with monitoring and evaluation and that may be used in national guidance development processes or in policy development processes - Policy dialogue (stakeholder dialogue[5]): systematically planned processes organized at the national or sub-national level to solicit the views, experiences and tacit knowledge of those who will be involved in or affected by decisions about appropriate options for addressing a health system problem in that specific setting, and a summary of which may be used in policy development processes - Evidence-Informed Policy Network (EVIPNet): A partnership among policymakers, stakeholders (including civil society) and researchers that promotes the systematic use of research evidence in policymaking about health systems. While focused typically at the national level and occasionally at the sub-national, EVIPNets are embedded within a regional and global collaborative social network supported by WHO[16,17] |
| --- |
